# The Functions and Mechanisms of Low-Level Laser Therapy in Tendon Repair (Review)

**DOI:** 10.3389/fphys.2022.808374

**Published:** 2022-02-15

**Authors:** Kexin Lyu, Xueli Liu, Li Jiang, Yixuan Chen, Jingwei Lu, Bin Zhu, Xinyue Liu, Yujie Li, Dingxuan Wang, Sen Li

**Affiliations:** ^1^Institute of Physical Education, Southwest Medical University, Luzhou, China; ^2^Spinal Surgery Department, The Affiliated Traditional Chinese Medicine Hospital of Southwest Medical University, Luzhou, China

**Keywords:** low-level laser therapy, tendon repair, tendinopathy, mechanism, function

## Abstract

Tendon injury is a common disease of the musculoskeletal system, accounting for roughly 30%–40% of sports system disorder injuries. In recent years, its incidence is increasing. Many studies have shown that low-level laser therapy (LLLT) has a significant effect on tendon repair by firstly activating cytochrome C oxidase and thus carrying out the photon absorption process, secondly acting in all the three phases of tendon repair, and finally improving tendon recovery. The repair mechanisms of LLLT are different in the three phases of tendon repair. In the inflammatory phase, LLLT mainly activates a large number of VEGF and promotes angiogenesis under hypoxia. During the proliferation phase, LLLT increases the amount of collagen type III by promoting the proliferation of fibroblasts. Throughout the remodeling phase, LLLT mainly activates M2 macrophages and downregulates inflammatory factors, thus reducing inflammatory responses. However, it should also be noted that in the final phase of tendon repair, the use of LLLT causes excessive upregulation of some growth factors, which will lead to tendon fibrosis. In summary, we need to further investigate the functions and mechanisms of LLLT in the treatment of tendon injury and to clarify the nature of LLLT for the treatment of diverse tendon injury diseases.

## Introduction

In recent years, researchers report that the prevalence of tendon injuries continues to rise, with young people being the most vulnerable group, and rotator cuff muscles and the Achilles tendon being the most common sites of injury (Xu and Murrell, 2008; Thomopoulos et al., 2015; Genc et al., 2020). It is generally thought that the primary cause of tendon injury is overuse, which not only alters the tendon structure but also causes many negative reactions such as tendon swelling, irregular collagen arrangement, and an increase in pathological molecules (Xu and Murrell, 2008; Kaux et al., 2011; Sereysky et al., 2013; Aicale et al., 2018).

Currently, tendon injuries are treated with a comprehensive range of treatments, which include conservative treatments (such as ultrasound, shock wave, platelet rich plasma, and low-level laser therapy, LLLT), surgery, and specific exercises to help with rehabilitation. Over the past decade, LLLT use has been increasingly examined in many clinical studies. It has been used to treat tendon injuries, with excellent results in tendon repair (Sereysky et al., 2013; Lipman et al., 2018). LLLT, also known as photobiomodulation, primarily reduces the degree of tendon injury by topical application of short-wavelength monochromatic light. The mechanisms of LLLT are mainly related to cytochrome C oxidase, and its functions include the promotion of angiogenesis, the acceleration of cell proliferation, the promotion of metabolism, and the release of inflammatory factors (Nogueira Junior and Moura Junior, 2015; Wang et al., 2016).

Most recent studies have been able to explain the overall mechanisms of LLLT in the treatment of tendon lesions and its treatment advantages. However, because LLLT’s mechanisms involve a large number of biomolecular changes and the interactions between them, there are still some unclear points in the study of its therapeutic mechanisms (Andarawis-Puri et al., 2015; Lopes Silva et al., 2020). Consequently, considering the significant impact of LLLT, this review will analyze the various effects of LLLT on tendon repair, explain its mechanisms of action in different stages of repair, and then present a discussion of its potential therapeutic use going forward. The overall aim of this review is to provide evidence for the future treatment of tendon lesions with LLLT and to explain more fully the explicit role of LLLT in the healing process.

### Search Strategy

**Figure 1:**
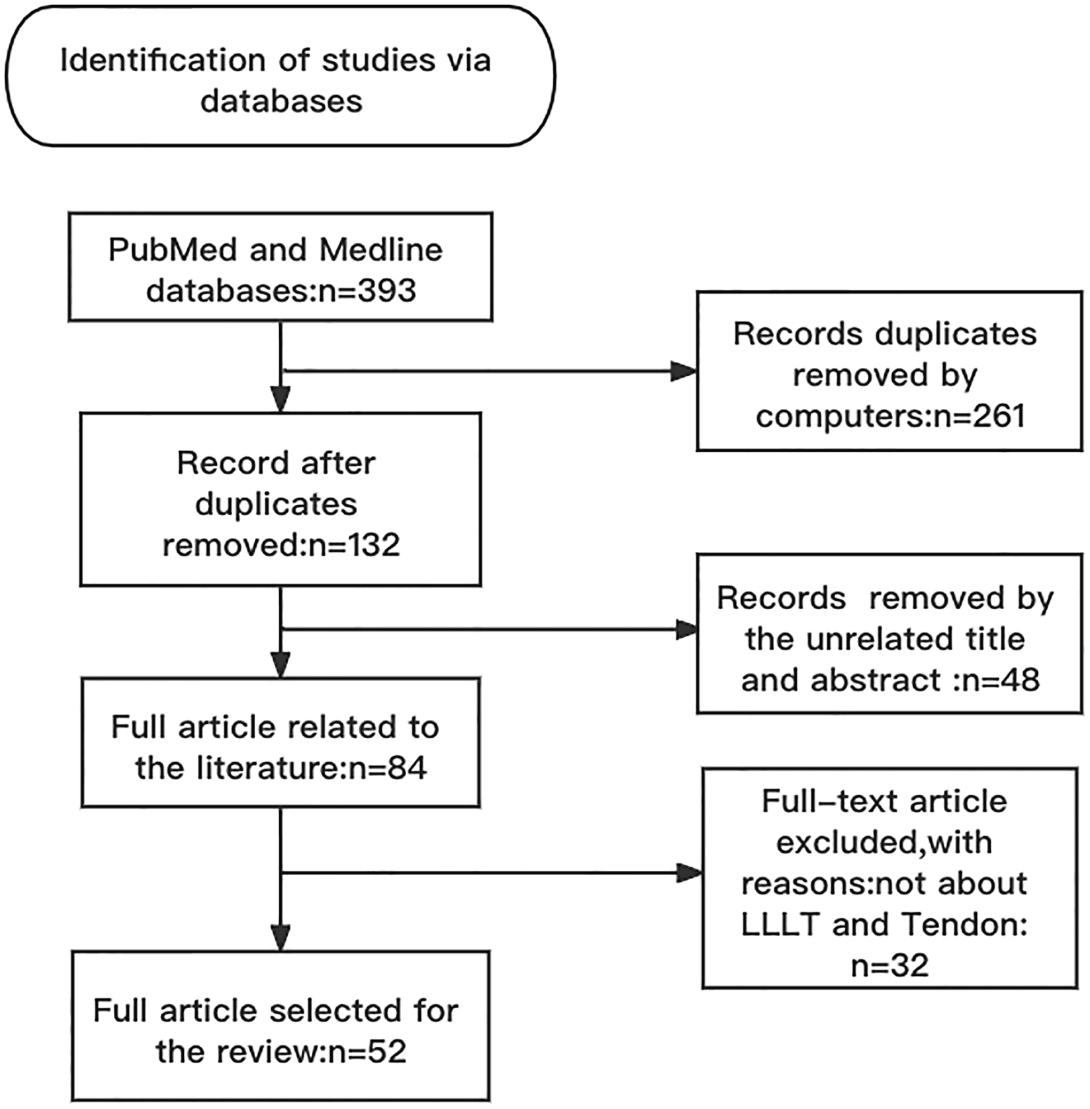
Article retrieval flow chart with inclusion and exclusion process.

(i) Search site: articles are from PubMed, a database of papers on biomedical science. (ii) Database: MEDLINE. (iii) Keywords: LLLT, tendon repair, tendinopathy, mechanism. (iv) Boolean algorithm: (LLLT OR photobiomodulation) AND (Tendinopathy OR Tendon injuries OR Tendon repair) AND function AND mechanism. (v) Retrieving timeframe: we searched the selected journals published from 2000 to 2021. (vi) Inclusion and exclusion criteria: articles were included if the topic is related to LLLT and tendon repair, while the article type was a review or an experimental paper. The retrieval process is shown in Figure 1.

## Characteristics and Merits of LLLT for Tendon Injuries in Clinical Practice

There are multiple options for treating tendinopathy that can be divided into surgical and non-surgical treatments. Tendon injuries that warrant operative treatments can be of two types. Firstly, for more severe tendon injuries, surgical therapies as described below are frequently performed, for instance, open debridement, tendonectomy, and tendon grafting, which allow for the removal of degenerative tissue in such a way as to stimulate tendon healing (Andarawis-Puri et al., 2015; Li and Hua, 2016). Secondly, a few tendon impairments that do not respond well to conservative treatment may still necessitate a surgical intervention (Alfredson and Cook, 2007).

The tendon disorders that demand non-operative remedies can also be illustrated in two categories. Primarily, for less symptomatic injuries, we commonly apply conservative treatments such as proper rest, Non-steroidal anti-inflammatory drugs (NSAIDs), injections, cryotherapy, physical therapy, etc. The typical modalities are as follows. NSAIDs have been widely used in clinical practice, but due to their innumerable side effects and lack of therapeutic efficacy, they are no longer used as a preferred treatment modality (Andres and Murrell, 2008). At present, the most respected method in non-surgical treatment is injection therapy, which includes platelet-rich plasma injections, mesenchymal stem cells (MSCs), hyaluronic acid injections, or other injectable therapies. Numerous studies have now proven the superiorities of using MSCs in tendon healing and perhaps in broader applications (Ragni et al., 2021). With numerous origins of MSCs, adipose-derived mesenchymal cells (ASCs) are optimal for inclusion in non-surgical protocols since they promote tendon repair more effectively than bone marrow MSCs (BMSCS). Firstly, ASCs are conveniently isolated from adipose tissue and are less susceptible to destruction. Secondly, they downregulate relevant inflammatory factors, enhance fibroblast proliferation, accelerate tendon cell differentiation, and boost angiogenesis. Finally, they further spur tissue regeneration and boost the process of tendon repair (Shen et al., 2020; Piccionello et al., 2021). While MSCs have considerable benefits in musculoskeletal disorders, there is still a certain risk of infection during the healing process. The effectiveness of MSCs have mostly been verified through animal studies; hence, we still require a wealth of *in vivo* trials to demonstrate the effectiveness of ASCs therapy (Sandona et al., 2021).

**Figure 2:**
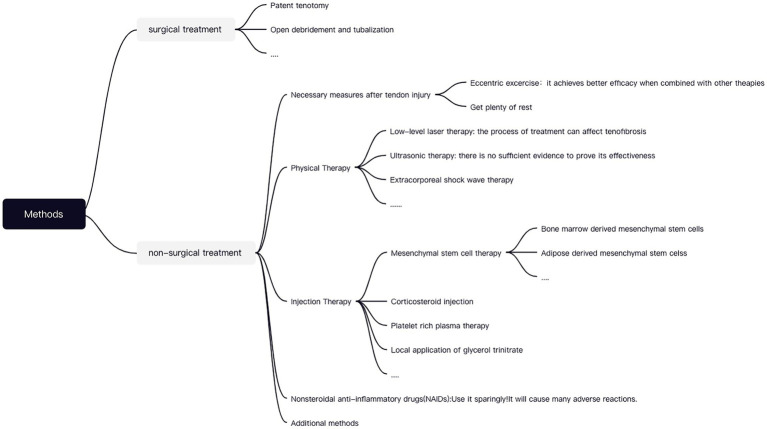
Different treatments for tendon injuries.

Next, postoperative rehabilitation to restore tendon function will be vital, and thus we will rely on certain nonoperative measures to assist in the rehabilitation process. For problems that will arise during postoperative rehabilitation (such as joint stiffness and muscle atrophy), physical therapy and massage can be utilized in conjunction with centrifugal exercises for training. Physical therapies that have similarities to LLLT include extracorporeal shock wave therapy, ultrasound therapy, and others. Among which, ESWT works by delivering energy deep into the tendon tissue. Studies have shown that ESWT in combination with other therapies (eccentric exercise) can substantially reduce the degree of injury (Stania et al., 2019). On the whole, there are several options for addressing tendon injuries, paradoxically, extensive experiments are still warranted to prove its availability (including *in vitro* and *in vivo* tests). Different treatments for tendon injuries are shown in Figure 2.

LLLT has been more widely used in clinical practice. Generally, the effect of LLLT varies with the duration of laser exposure, which invariably involves the biological effects of photobiomodulation (Andres and Murrell, 2008; Fortuna et al., 2018). However, depending on the duration of laser irradiation, its effects on the deleterious sites keep diverse. The short-term effect is that an irradiation lasting a few seconds or minutes will upregulate the ATP content (Clijsen et al., 2017). The long-term effect is that the tendon repair process will be in a phase of cell proliferation after prolonged laser exposure, which accelerates the proliferation of fibroblasts and then promotes the synthesis of collagen type I and III (Fortuna et al., 2018). In clinical practice, nevertheless, there are still no specific treatment parameters or treatment protocols for the diverse array of tendon injuries (Bjordal et al., 2006). Much of the research has examined that when the damaged area receives near-infrared light with a length of 600–1,000 nm and a radiation value of 3–10 J/cm^2^, it normally promotes tendon repair to a large extent. In the case of tendon injuries requiring operative therapy, the use of LLLT is typically placed after surgical treatment. First of all, a slice of open tendon injuries are often treated with a laser within 4 J/cm^2^, while in contrast, for quite a few closed injuries (degenerative lesions), a laser in the range of 10–50 J/cm^2^ should be used (Zein et al., 2018).

## The Basis of How LLLT Works: C Cytchrome C Oxidase

**Figure 3:**
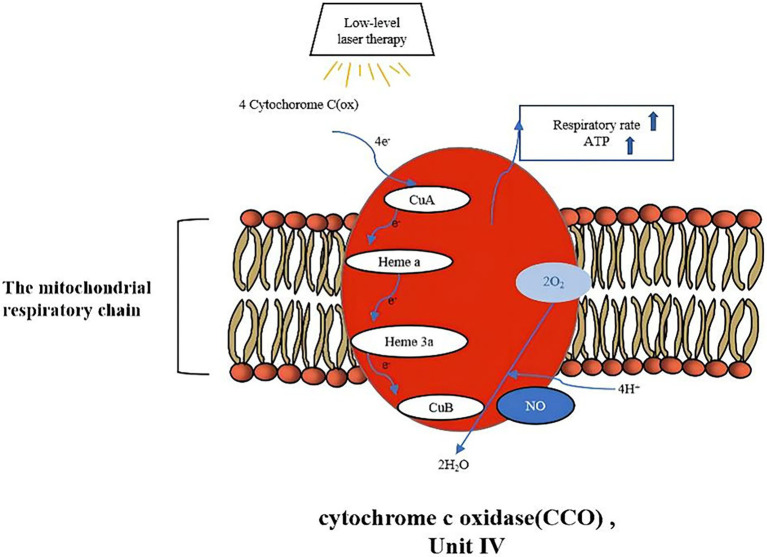
The absorption of photons.

Since the low-level laser treatment process is a photochemical reaction, it is closely related to the absorption of photons, which are associated with cytochrome C oxidase (CCO). LLLT will improve ATP synthesis and respiration rate through this process, thus promoting tendon injury repair. Conversely, laser therapy does not work when CCO activation fails (Wang et al., 2016). CCO, as an enzyme at the end of the mitochondrial respiratory chain, mainly performs electron transfer in the process of energy metabolism and promotes REDOX reaction under the condition of cellular hypoxia. Under normal conditions, CCO not only oxidizes the four reduced cytochrome C molecules but also produces four protons, which combine with oxygen to form water and activate ATPase to produce large amounts of ATP. Nitric oxide (NO) can bind to CuB to inhibit this process (Farivar et al., 2014). However, when the injured tissue is irradiated by laser, LLLT can considerably improve CCO activity and enhance the oxidative metabolism of cells (Farivar et al., 2014; Clijsen et al., 2017; Tsai and Hamblin, 2017; Hamblin, 2018), NO can be dissociated, so the respiration rate will increase, and a large amount of ATP will be generated. Consequently, to maintain a balance between oxygen intake and demand, cellular metabolism is improved, and the hemodynamic changes, thus promoting healing of tendons (Farivar et al., 2014; Wang et al., 2016; Tsai and Hamblin, 2017). The absorption of photons is illustrated in Figure 3.

## Mechanisms of Action of LLLT in the Three Phases of Tendon Repair

**Figure 4:**
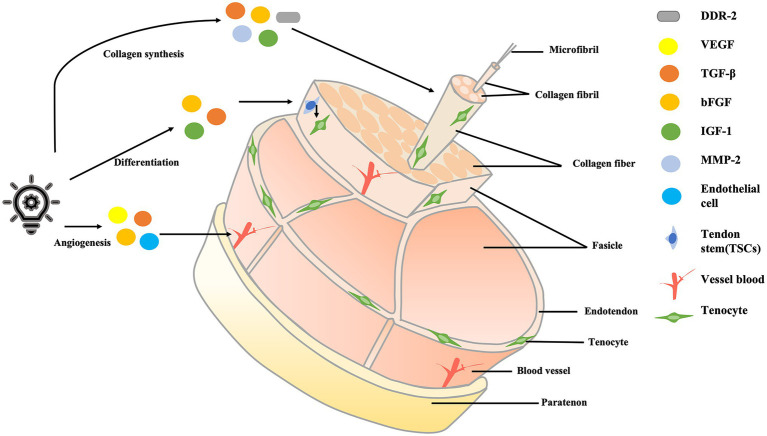
The mechanisms of LLLT in the treatment of tendon injury. LLLT, low-level laser therapy.

The process of tendon healing can be divided into three main phases: the inflammatory phase, the cell proliferation phase, and the tendon shaping phase. The inflammatory phase occurs within 48 h of tendon injury—first, blood clots fill the injured tissue, and then fibrin continues to attach to the damaged tissue as inflammation develops (Lopes Silva et al., 2020). The proliferative phase is mainly characterized by the formation of large amounts of granulation tissue, including the proliferation of fibroblasts and the synthesis of type III collagen. The remodeling phase is characterized by a remodeling of the ECM, which is not only accompanied by a significant reduction or apoptosis of cells but also by a reduction of type III collagen and the promotion of type I collagen synthesis (Andarawis-Puri et al., 2015). The mechanisms of LLLT in the treatment of tendon injury are shown in Figure 4. The factors associated with ECM remodeling are shown in Table 1.

**Table 1:** LLLT regulates the remodeling of ECM.

The remodeling of ECM	Factors associated with the process
Protein synthesis	TGF-β, MMP-2, MMP-3, and MMP-14
Protein degradation	TGF-β, MMP-2, MMP-3, MMP-9, MMP-13, and MMP-14

*ECM, extracellular matrix; TGF-β, transforming growth factor-β; MMP-2, matrix metalloprotease 2; MMP-3, matrix metalloprotease 3; MMP-9, matrix metalloprotease 9; MMP-13, matrix metalloprotease 13; and MMP-14, matrix metalloprotease 14*.

### LLLT Promotes Angiogenesis During the Inflammatory Phase

During the inflammatory phase of tendon repair, LLLT primarily promotes angiogenesis. In general, tendon injury is associated with a series of pathological changes, such as vascular infiltration and upregulation of vascular endothelial growth factors (VEGF; Xu and Murrell, 2008). The mechanisms of LLLT in the treatment of tendon injury are mainly mediated by photobiological stimulation rather than by thermal effect (Rand et al., 2007; Lin et al., 2010). Firstly, the necessary condition for LLLT to promote angiogenesis is hypoxia, and secondly, LLLT can regulate the activity of angiogenic factors. Angiogenesis is mainly associated with hypoxia inducible factor 1α (HIF-1α) activation, large expression of VEGF, and downregulation of matrix metallopeptidase 2 (MMP-2; Hamblin, 2018).

The hypoxia-inducible factor-HIF-1α plays an important role in the process of tendon tissue facing hypoxia because hypoxia is a necessary condition for the formation of new blood vessels. Since the absorption of photons is accompanied by an increase in respiration rate, the amount of oxygen in the tissue drops sharply, thereby activating HIF-1α (Hamblin, 2018).

Hypoxia causes an increase in the number of growth factors. The factor most associated with angiogenesis is vascular endothelial growth factor (VEGF), which has many effects on vascular endothelial, including promoting the establishment of channels between the neovascularization and circulation systems, accelerating the proliferation and differentiation of endothelial cells, and increasing vascular permeability (Adabbo et al., 2016; Lopes Silva et al., 2020).

Therefore, to meet the requirements of cell oxygenation after the use of LLLT, a series of pro-angiogenic factors (such as VEGF) increase and combine with other cell receptors, the process activates the newly created cells, which then generate new blood vessels. This process achieves the purpose of promoting hemodynamic changes, improving angiogenesis, and tendon injury recovery (Alves et al., 2014; de Freitas and Hamblin, 2016; Wang et al., 2016; Fortuna et al., 2018). While this mechanism has been proposed in much of the literature, experimental evidence is still needed to validate the process. As shown in Table 2, LLLT can modulate different cytokines during the injury and repair phases.

**Table 2:** LLLT downregulates diverse inflammatory cytokines.

	Different cytokines associated with an inflammatory response
Damage stage	M1 macrophages, neutrophils, TNF-α, IL-6, IL-1β, PGE2, Cox-2, and NF-kB pathway
Repair phase	M2 macrophages, Cox-7, and IL-10

*LLLT, low-level laser therapy; TNF-α, tumor necrosis factor-α; IL-6, interleukin-6; IL-1β, interleukin-1β; PGE2, prostaglandin E2; Cox-2, cyclooxygenase 2; NF-kB pathway, nuclear factor kappa-B; Cox-7, cyclooxygenase 7; and IL-10, interleukin-10*.

### LLLT Promotes the Synthesis of Collagen During the Cell Proliferation Phase

The proliferation phase is often accompanied by the formation of a large number of collagen fibers, which are closely related to collagen levels. LLLT can primarily promote collagen synthesis by promoting fibroblast proliferation, which improves the ability of the tendon to heal. Fibroblast activity is mainly related to a multifunctional growth factor-- transforming growth factor-β (TGF-β; Luo et al., 2013). TGF-β is the most effective pro-fibrosis factor in the process of tendon repair and is directly related not only to wound healing but also to scarring during tendon repair; this factor also reduces the number of senescent cells (Sereysky et al., 2013). TGF-β has two main functions. Firstly, it promotes wound healing and scar formation, and secondly, TGF-β plays a key role in muscle fibrosis because it affects changes in ECM-degrading proteases (Luo et al., 2013; Sereysky et al., 2013; Delaney et al., 2017). LLLT can reduce TGF-β content after tendon injury, thus reducing the probability of tendon fibrosis and a series of complications such as tendon tearing after surgery, while also indirectly promoting collagen synthesis (Assis et al., 2013; Delaney et al., 2017). Therefore, LLLT treated muscles have not evidenced excessive scar formation during the repair process and have also shown a large number of regenerating muscle fibers (Luo et al., 2013).

Additionally, fibroblast proliferation is closely related to the collagen receptor—discoidindomainreceptor2 (DDR2), which is regulated by MMP-2. DDR2 collagen receptors regulate fibroblast proliferation and promote ECM synthesis, which is important for tendon healing (Illescas-Montes et al., 2019). MMP-2 is an important molecule involved in the regulation of collagen protein synthesis and degradation. LLLT, through the gene expression of MMP-2, enhances its activity and improves the probability of combination with DDR collagen receptors, thereby minimizing damage to the accumulation of collagen, promoting the synthesis of collagen, and improving tendon healing (Abate et al., 2009; Adabbo et al., 2016).

After the proliferation of fibroblasts, collagen synthesis will be affected. A large amount of collagen can be synthesized into collagen fibrils, which are bundled into collagen fibers (Galloway et al., 2013; Wu et al., 2017). After the formation of collagen fibers, the mechanical strength of the tendon can be greatly improved and the tension resistance of injured tissue will also be enhanced, thus indirectly promoting the synthesis of collagen, thus effectively affecting the efficacy of tendon healing (Lock Silveira et al., 2013; Wu et al., 2017).

Studies comparing the diversity of collagen levels after treating tendinopathy with LLLT have shown that the use of LLLT can maximize the contents of type I and III collagen (Lopes Silva et al., 2020). It should be noted, however, that different frequency laser treatments have different effects on tendon repair, among which low-frequency pulsed laser treatment has been shown to maximize the synthesis effect of type I protein, thus regulating the generation of collagen fiber (Guerra et al., 2013).

### LLLT Reduces Inflammatory Response During the Tendon Shaping Phase

In general, tendon injury is associated with cytokines such as tumor necrosis factor-α (TNF-α), Interleukin-1β (IL-1β), and Interleukin-10 (IL-10). In the treatment of tendinopathy, LLLT can effectively reduce the content of pro-inflammatory cytokines and also regulate the mRNA expression of anti-inflammatory cytokines (Pires et al., 2011). In the trauma stage, mitochondria are key organelles for activating inflammatory macrophages, which can stimulate M1 macrophages and neutrophils, and produce cytokines such as COX-2, TNF-α, and IL-1β, then activate MAPK and NF- kB pathways, and finally lead to tendon inflammation (Lopes Silva et al., 2020). COX-2 affects the conversion of arachidonic acid into prostaglandins. After 48 h, tendon injury enters the repair stage, during which LLLT can decrease the expression of the NF-kB gene, reduce the activity of COX-2, lower the number of inflammatory mediators and pro-inflammatory factors, and activate M2 macrophages to release anti-inflammatory factors, thus producing anti-inflammatory effects and promoting tendon repair (Rodrigues et al., 2014; Fortuna et al., 2018).

Among these factors, LLLT has a significant effect on TNF-α and IL-6 (Pires et al., 2011; Klatte-Schulz et al., 2018). This is because IL-6 and TNF-α are key cytokines in the development of tendon diseases, and their expressions change with the changes of the tendon (Morita et al., 2017). IL-6 plays a central role in the early stages of tendon injury or after prolonged stress on healthy tendons, and its secretion tends to increase (Legerlotz et al., 2012; Morita et al., 2017). In contrast, LLLT therapy has been shown to significantly reduce the production of pro-inflammatory cytokines other than IL-1β, especially IL-6 mRNA expression (Pires et al., 2011).

Laser treatment at 660 or 870 nm can significantly reduce the expression of TNF-α and IL-6 mRNA, which can significantly reduce the degree of tendon fibrosis and stiffness, maximize the growth capacity of fibroblasts, and improve contraction after tendon injury, thus improving the repairability of muscles (Ryan and Smith, 2007; Mesquita-Ferrari et al., 2011; Pires et al., 2011).

## Conclusion and Perspectives

Tendon injury is a series of muscular imbalances caused by muscle overstrain or poor treatment at the beginning of the disease. Overloading can lead to partial tearing of the tendon initially, and tendon tears are often accompanied by some inflammation and degeneration of the tendon. If not treated in a timely fashion, it will cause structural imbalance and tendon tears, and other consequences (Galloway et al., 2013).It is essentially an unsuccessful healing process, primarily because the inflammatory response destroys the probability of damage repair and is accompanied by several biological changes.

At present, most studies have shown a positive effect of LLLT on tendon repair, especially on some biological factors and structural components in terms of anti-inflammation and analgesia, but this is also dependent on the parameters of the treatment (Bjordal et al., 2006; Lopes Silva et al., 2020). LLLT, also known as photoluminescence, is a non-invasive method that increases the ability to heal part of the damage and to enhance tissue repair without overheating the tissue as the infrared light used in the treatment is not transmitted by an external device, but rather by the body’s heat to drive some of the materials to emit infrared to the damaged area (Tsai and Hamblin, 2017; Wickenheisser et al., 2019; Dompe et al., 2020). The process of using LLLT to repair damaged tendons is mainly to exert non-thermal and photochemical reactions in cells to treat the damaged structures. For patients, near-infrared light is commonly used, and LLLT can play its full role under this condition (Wickenheisser et al., 2019). LLLT treatment mainly affects the activity of mitochondria in cells, increasing ATP content, the change of ROS species, and the expression of biological factor mRNA, to stimulate tendon healing (Farivar et al., 2014). Studies have shown that the use of LLLT, combined with certain exercise therapy such as centrifugal exercise and isometric contraction, can treat tendinopathy to a greater extent than other existing therapies (Jang and Lee, 2012; Girgis and Duarte, 2020).

Based on clinical and animal studies, the mechanism of LLLT promoting tendon injury repair involves reducing the production of inflammatory factors, accelerating the release of anti-inflammatory factors, and promoting angiogenesis. In the phase of promoting angiogenesis, LLLT mainly promotes the production of a series of factors related to angiogenesis through the activation of hypoxia-inducible factors, activates a series of washing, and restores blood function (Xu and Murrell, 2008; Lin et al., 2010; Alves et al., 2014; de Freitas and Hamblin, 2016; Wang et al., 2016; Fortuna et al., 2018; Hamblin, 2018). During the remodeling phase of ECM, LLLT is mainly related to TGF-β and matrix metalloproteinases, and these factors are closely related to the proliferation of fibroblasts, so LLLT regulates the content of different biomolecules, thus promoting collagen synthesis (Mesquita-Ferrari et al., 2011; Assis et al., 2013; Luo et al., 2013; Sereysky et al., 2013; Alves et al., 2014; Delaney et al., 2017; Illescas-Montes et al., 2019). In terms of anti-inflammation, LLLT mainly reduces the expression of the NF-KB gene and COX-2 activity to release a large number of anti-inflammatory factors, so as to achieve the goal of tendon repair (Pires et al., 2011; Deng et al., 2012; Rodrigues et al., 2014; Fortuna et al., 2018). The key factors associated with tendon repair are shown in Table 3.

**Table 3:** The role of diverse biomolecules.

Different biological factors	Function
TNF-α	Effective pro-inflammatory cytokines
TGF-β	Factors involved in wound healing
IL-6	Key molecules in the early stages of tendon injury and after stress on healthy tendons
MMPs, TIMPs	The ratio is especially essential, and it has a great influence on the formation of tendon collagen
VEGF	Vascular endothelial growth factor plays an important role in blood regulation
NF-κB pathway	Nuclear factor kappa-β, it has a huge impact on the inflammatory response and immune response of cells

*TNF-α, tumor necrosis factor-α; TGF-β, transforming growth factor-β; IL-6, interleukin-6; MMPs, matrix metalloprotease; TIMPs, tissue inhibitors of metalloproteinases; VEGF, vascular endothelial growth factor; and NF-kB, nuclear factor kappa-β*.

Few studies to date have provided an assessment of the degree of tendon fibrosis after the use of LLLT. TGF-β is the most potent pro-fibrotic factor in the process of tendon healing, and it is directly related to the late scar formation process (Sereysky et al., 2013). While the use of LLLT can inhibit the activity of TGF-β, there have been no definitive studies on LLLT use in this regard. Therefore, it is more likely to cause tendon scarring. Furthermore, the parameters for the use of LLLT present a challenge that continues to be tackled. Presently, there are few clinical criteria for specific conditions, thus further experiments have to be conducted to verify the application of the laser (Lipman et al., 2018).

As incidents of tendon injury are becoming more common, and no definitive, gold standard treatment for tendinopathy has been developed, animal models, despite their differences to humans, will be essential for future research. In the process of using laser treatments for future research, it will be important to strictly compare the differences between control groups and the experimental groups and to accurately control and monitor the frequency levels of low-frequency lasers (Shepherd and Screen, 2013; Hast et al., 2014). In any future study of LLLT, we also need to better ascertain the therapeutic properties and usage specifications of lasers, and further explore the process of LLLT in accelerating cell metabolism, so as to improve the use and efficacy of LLLT in treating tendon injuries.

## Author Contributions

KL, XuL, and JL designed the present manuscript. KL drew the manuscript. YC, LJ, BZ, YL, and XiL performed a literature search and selected the studies to be performed. KL, DW, and SL revised the manuscript. All authors contributed to the article and approved the submitted version.

## Conflict of Interest

The authors declare that the research was conducted in the absence of any commercial or financial relationships that could be construed as a potential conflict of interest.

## Publisher’s Note

All claims expressed in this article are solely those of the authors and do not necessarily represent those of their affiliated organizations, or those of the publisher, the editors and the reviewers. Any product that may be evaluated in this article, or claim that may be made by its manufacturer, is not guaranteed or endorsed by the publisher.
